# MSDTCN-Net: A Multi-Scale Dual-Encoder Network for Skin Lesion Segmentation

**DOI:** 10.3390/diagnostics15222924

**Published:** 2025-11-19

**Authors:** Da Li, Xinyang Wu, Qin Wei

**Affiliations:** School of Information Engineering, Wuhan University of Technology, Wuhan 430070, China; frankli26@whut.edu.cn (D.L.); 270376@whut.edu.cn (X.W.)

**Keywords:** skin lesion segmentation, convolutional neural networks, transformer, multi-scale receptive field, hierarchical feature transfer

## Abstract

**Background/Objectives:** Accurate segmentation of skin lesions is essential for early skin cancer detection. However, traditional CNNs are limited in modeling long-range dependencies, leading to poor performance on lesions with complex shapes. **Methods:** We propose MSDTCN-Net, a dual-encoder network that integrates ConvNeXt and Deformable Transformer to extract both local details and global semantic information. A Squeeze-and-Excitation (SE) mechanism is introduced to adaptively emphasize important channels. To address scale variation in lesions, we design a Multi-Scale Receptive Field (MSRF) module combining multi-branch and dilated convolutions. Furthermore, a Hierarchical Feature Transfer (HFT) mechanism is employed to guide high-level semantics progressively to shallow layers, enhancing boundary reconstruction in the decoder. **Results:** Extensive experiments on the ISIC 2016, ISIC 2017, ISIC 2018, and PH2 datasets show that MSDTCN-Net achieves competitive performance across metrics including IoU, Dice, and ACC, validating its effectiveness and generalization in skin lesion segmentation. **Conclusions:** MSDTCN-Net effectively combines local and global feature extraction, multi-scale adaptability, and semantic guidance to achieve high-accuracy skin lesion segmentation, demonstrating its potential in clinical diagnostic applications.

## 1. Introduction

Skin cancer is one of the most common types of cancer worldwide, and its incidence has steadily increased over the past few decades. According to the 2020 global cancer statistics from the World Health Organization (WHO), cancer has become one of the leading causes of death globally, with nearly 10 million deaths from cancer in 2020. Non-melanoma skin cancer (NMSC) accounts for approximately 1.2 million new cases annually, making it one of the most prevalent cancer types globally. Although melanoma represents only a small portion of all skin cancer cases, its biological behavior is highly aggressive, and it is the leading cause of death related to skin cancer. According to statistics from the American Cancer Society (ACS), when melanoma is detected early and treated promptly, the five-year survival rate for patients can exceed 99%. However, if cancer cells have spread to distant organs, the five-year survival rate may drop to 32%.

Dermoscopy is a non-invasive imaging technique that enhances the visibility of skin lesions by reducing surface light reflection and uniformly illuminating the area [1]. However, dermoscopy diagnosis relies on clinicians’ subjective judgment, making it susceptible to factors like lighting changes, lesion complexity, and hair interference, leading to diagnostic instability. This highlights the potential of computer-aided diagnosis (CAD) systems for automatic skin lesion analysis [2]. Automatic segmentation is crucial in CAD to extract lesion areas accurately; however, it faces challenges such as diverse lesion shapes, indistinct boundaries, low contrast, and imaging artifacts. Traditional segmentation methods, like thresholding [3], clustering [4], and region merging [5], rely on shallow features but struggle with generalizability and adapting to the complexity of skin lesions.

In recent years, deep learning has greatly advanced medical image segmentation. U-Net and its variants [6] remain strong baselines because the encoder–decoder topology with skip connections enables multi-scale feature fusion. Nevertheless, their locality-dominated receptive fields often struggle with blurred boundaries, low contrast, and irregular lesion geometry. To mitigate these issues, multi-scale CNN designs typified by Atrous Spatial Pyramid Pooling in the DeepLab [7] enlarge the effective receptive field through parallel dilated convolutions, thereby enriching context while preserving resolution. At the same time, Transformer-based models [8] have been introduced to capture long-range dependencies. TransFuse [9] combines a convolutional branch and a Transformer branch and fuses them bidirectionally so that local texture cues and global semantic relations can be aligned across stages. Swin-UNETR [10] adopts a Swin-Transformer encoder with shifted windows to realize hierarchical self-attention at multiple scales and couples it with a UNet-style decoder for dense prediction. MedT [11] employs a gated axial attention mechanism with learnable positional-encoding gates and introduces a Local–Global training scheme that combines a global full-image branch with a local patch branch, enabling the model to capture long-range dependencies effectively under limited medical data conditions. Building on these trends, hybrid CNN–Transformer systems and dual-encoder frameworks decouple local detail from global semantics and then reunify them along the decoding path.

In this paper, we propose a novel dual-encoder segmentation network, MSDTCN-Net, specifically designed for skin lesion segmentation tasks. We conduct extensive experiments on four publicly available datasets: ISIC 2016 [12], ISIC 2017 [13], ISIC 2018 [14, 15], and PH2 [16]. Comparative experiments with current state-of-the-art segmentation methods demonstrate the effectiveness of the proposed approach. Our main contributions are summarized as follows:

(1) We propose a dual-encoder architecture to balance the ability to extract local features and model long-range dependencies. ConvNeXt [17] is responsible for capturing local detail information, while Deformable Transformer [18] enhances the modeling of long-range dependencies through its deformable attention mechanism.
(2) We introduce the Squeeze-and-Excitation (SE) [19] mechanism and Multi-Scale Receptive Field (MSRF) module to enhance feature fusion capabilities and improve the model’s adaptability to lesions of different scales.
(3) We propose the Hierarchical Feature Transfer (HFT) mechanism and optimize the decoder structure to improve boundary recovery for lesion areas.

## 2. Related Works

Deep learning has made significant progress in the field of medical image segmentation, with U-Net being one of the most representative Convolutional Neural Network (CNN) architectures. However, due to the blurred boundaries and low contrast of many skin lesions, the original U-Net often struggles to accurately segment lesion areas in certain cases. As a result, many researchers have proposed several improvements to U-Net, resulting in various network variants. For example, U-Net++ [20] enhances feature fusion through dense skip connections, improving segmentation accuracy; ResU-Net [21] incorporates residual connections to mitigate the vanishing gradient problem, enhancing the network’s training stability; AttU-Net [22] introduces attention mechanisms in the decoder to focus the model’s attention on key regions; and DCSAU-Net [23] combines depthwise separable convolutions with adaptive attention to improve computational efficiency and boundary segmentation capabilities. While these U-Net variants have made improvements in medical image segmentation, the limitations of CNN’s local receptive fields still persist, making it difficult for the model to capture global information across distant pixels.

To address the limitations of CNNs in capturing global information, researchers have introduced the Transformer self-attention mechanism to model long-range dependencies. Transformer, through global self-attention computation of input features, effectively models the relationships between distant pixels, making it particularly well-suited for medical image segmentation tasks involving complex morphological structures. The success of Vision Transformer (ViT) [24] in image classification tasks has driven Transformer-based research in the medical image segmentation field. Swin Transformer [25], an improved version of the Vision Transformer, adopts a sliding window (Shifted Window) mechanism, which reduces computational complexity while enhancing local information modeling.

With the widespread application of Transformer in medical image segmentation, researchers have further explored hybrid architectures that combine CNNs and Transformers to leverage the strengths of CNNs in extracting local fine-grained features while taking advantage of Transformers’ capability in modeling long-range dependencies. For instance, TransAttUNet [26] uses Transformer Self-Attention (TSA) and Global Spatial Attention (GSA) modules to effectively extracts both deep and shallow features, improving segmentation performance in cases where lesion boundaries are complex and contrast is low. SUTrans-Net [27] employs a Spatial Group Attention (SGA) module to optimize the spatial attention mechanism, enhancing the feature representation of key region. FAT-Net [28] uses a dual-encoder structure and designs a Feature Adaptation Module (FAM) to match feature distributions of different layers in skip connections. DEU-Net [29] utilizes a dual-encoder U-Net structure, combining an EfficientNet-B6 convolutional encoder with a MaxViT Transformer encoder to simultaneously extract local details and global context. Pact-Net [30] designs a Channel-Space Fusion (CSF) module and a Self-Selection Multi-Scale Fusion (SSMF) module, optimizing feature fusion from different branches. DEMF-Net [31] integrates a dual encoder composed of a multi-scale Transformer branch and a CNN branch, and further introduces Dual-branch Attention Fusion (DAF), an Advanced Feature Fusion Module (AFFM), and Characterization Supplementary Blocks (CSBs) to reinforce edge details during decoding. DefNet [32] aggregates a convolutional distillation module (CDM) with a dual-branch attention transformer (DBAT) and employs a Multi-Scale Feature Enhancement Module (MFEM) to improve skip-level fusion. MEFP-Net [33] adopts an asymmetric dual-encoder in which a Global Information Extraction Module (GIEM) complements the main CNN pathway; its decoder employs a Multi-Scale Adaptive Feature Fusion Module (MAFFM) with attention to suppress background noise, followed by an Atrous Pooling Dense Perception Module (APDPM) that strengthens boundary perception.

## 3. Methods

**Figure 1:**
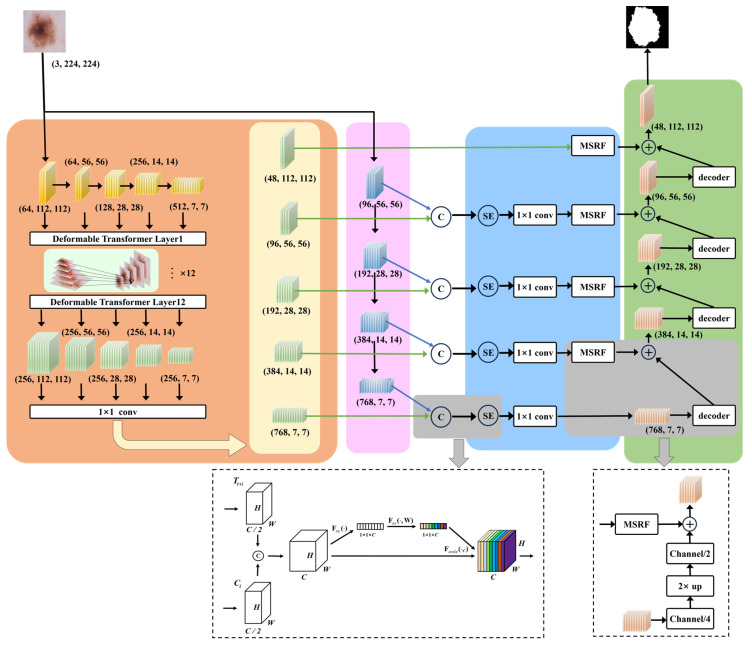
Overview of the proposed MSDTCN-Net architecture, consisting of a dual encoder with ConvNeXt and Deformable Transformer for feature extraction, SE mechanism and MSRF module for feature enhancement, and an HFT decoder for precise skin lesion segmentation. The symbol “+” denotes element-wise summation, and the symbol “C” denotes concatenation.

The overview of our proposed MSDTCN-Net is shown in Figure 1. This chapter mainly introduces the network’s overall architecture, including data preprocessing, feature extraction, feature enhancement, decoding, and the loss function. For data preprocessing, we employ a series of morphological operations to remove hair artifacts from the images, ensuring clearer inputs for segmentation. For feature extraction, we use a dual-encoder architecture, with ConvNeXt capturing local details and Deformable Transformer modeling long-range dependencies to enhance global context. For feature enhancement, we introduce the Squeeze-and-Excitation (SE) mechanism to perform channel-wise adaptive weighting, alongside the Multi-Scale Receptive Field (MSRF) module, which captures features across multiple scales using multi-branch and dilated convolutions. For decoding, we propose the Hierarchical Feature Transfer (HFT) mechanism that effectively restores spatial details by combining high-level semantic information with low-level features, improving segmentation accuracy. For the loss function, we adopt a hybrid loss function to improve the overall segmentation accuracy of the model, enhancing its performance in detecting lesion areas.

### 3.1. Preprocessing of Skin Lesion Images

Skin lesion images may suffer from issues like pixel intensity variations, and artifacts, which can affect segmentation accuracy. To address these, we designed a standardized image preprocessing pipeline.

**Figure 2:**
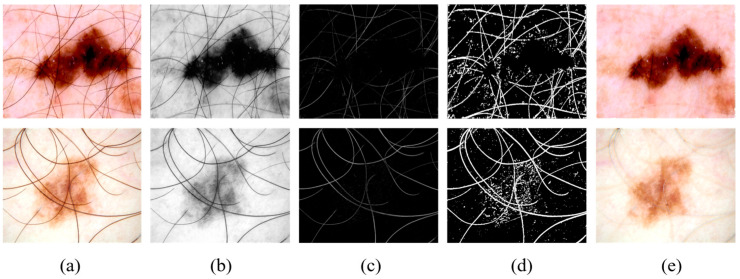
Visualization of the standardized image preprocessing steps, including (**a**) input images, (**b**) grayscale transformation results, (**c**) blackhat filtered images, (**d**) thresholded binary images, and (**e**) final inpainted images.

As shown in Figure 2a,b, the images are converted to grayscale to remove color information, focusing more on texture and structural features, simplifying the image complexity.

As shown in Figure 2b,c, a morphological-based black-hat transform is applied to remove hair artifacts, which are fine structures that can interfere with precise lesion segmentation.

As shown in Figure 2c,d, a thresholding operation is performed on the black-hat transformed image, generating a binary mask to highlight the hair artifact regions.

As shown in Figure 2d,e, the INPAINT_TELEA algorithm is used to repair the artifact regions, restoring the original structure and texture of the image.

To further enhance robustness, we also apply data augmentation, including bounded random rotations (±15°), random horizontal and vertical flips (each with probability 0.5), and mild adjustments to brightness, contrast, saturation, and hue within small ranges (0.03).

### 3.2. Dual-Encoder Feature Extraction

ConvNeXt is a deep optimization of traditional convolutional neural networks (CNNs). The core computation module, the ConvNeXt block, uses depthwise convolution for feature extraction and incorporates channel expansion and normalization operations to enhance feature learning ability. The specific computation process can be represented by Equation (1):

(1) $$X_{\mathrm{out}}=X_{\mathrm{in}}+W_{2}\sigma (W_{1}\mathrm{LN}(\mathrm{DWConv}(X_{\mathrm{in}})))$$

where $X_{\mathrm{in}}$ and $X_{\mathrm{out}}$ represent the input and output feature maps, DWConv(⋅) represents the 7 × 7 depthwise convolution, LN(⋅) represents the LayerNorm normalization, $W_{1}$ and $W_{2}$ are the weight matrices for the 1 × 1 pointwise convolutions, and σ(⋅) represents the GELU activation function. In our model, the input image $X\in {\mathbb{R}}^{3\times 224\times 224}$ is passed through the ConvNeXt encoder, resulting in four feature maps at different scales: $C_{1}\in {\mathbb{R}}^{96\times 56\times 56}$, $C_{2}\in {\mathbb{R}}^{192\times 28\times 28}$, $C_{3}\in {\mathbb{R}}^{384\times 14\times 14}$, $C_{4}\in {\mathbb{R}}^{768\times 7\times 7}$. These multi-scale features provide information ranging from low-level textures to high-level semantics, which facilitates subsequent feature fusion and lesion region segmentation.

The Deformable Transformer introduces a Deformable Attention mechanism with a sparse sampling strategy, allowing the model to adaptively select focus points and compute attention only at a few key locations in the feature map. To fully leverage multi-scale information, we employ ResNet34 as the backbone network to extract features at 5 different scales. To enhance the model’s ability to capture low-level boundary information, we retain the feature layers before the max-pooling operation to preserve more spatial details. These 5 feature maps are denoted as $R_{1}\in {\mathbb{R}}^{64\times 112\times 112}$, $R_{2}\in {\mathbb{R}}^{64\times 56\times 56}$, $R_{3}\in {\mathbb{R}}^{128\times 28\times 28}$, $R_{4}\in {\mathbb{R}}^{256\times 14\times 14}$, $R_{5}\in {\mathbb{R}}^{512\times 7\times 7}$. Due to the varying number of channels in these features, we apply 1 × 1 convolutions to unify the channel count across all feature maps for the Deformable Transformer, as shown in Equation (2):

(2) $$R_{i}^{\prime }={\mathrm{Conv}}_{1\times 1}(R_{i}),\;i\in \{1,2,3,4,5\},\;R_{i}^{\prime }\in {\mathbb{R}}^{256\times H_{i}\times W_{i}}$$

We input the aligned features into a 12-layer Deformable Transformer for multi-scale feature fusion, resulting in five sets of enhanced representations $T_{1}\in {\mathbb{R}}^{48\times 112\times 112}$, $T_{2}\in {\mathbb{R}}^{96\times 56\times 56}$, $T_{3}\in {\mathbb{R}}^{192\times 28\times 28}$, $T_{4}\in {\mathbb{R}}^{384\times 14\times 14}$, $T_{5}\in {\mathbb{R}}^{768\times 7\times 7}$.

### 3.3. Squeeze-and-Excitation Mechanism and Multi-Scale Receptive Field

The SE mechanism is applied to the fused features of C1 + T2, C2 + T3, C3 + T4, C4 + T5 to optimize the complementarity between local and global information. We first compute the global feature representation for each channel using Global Average Pooling. Then, we calculate the channel attention weights by passing the computed global channel information through two fully connected layers with reduction ratio r = 16, we perform channel weighting by applying the computed channel attention weights to the original input features. The final fused feature representation is shown in Equation (3):

(3) $$F_{i}^{SE}=f_{1\times 1}(z_{i}\cdot (C_{i}⊕T_{i+1}))$$

where ⊕ denotes the concatenation operation along the channel dimension, $z_{i}$ represents the channel attention weights computed by SE.

**Figure 3:**
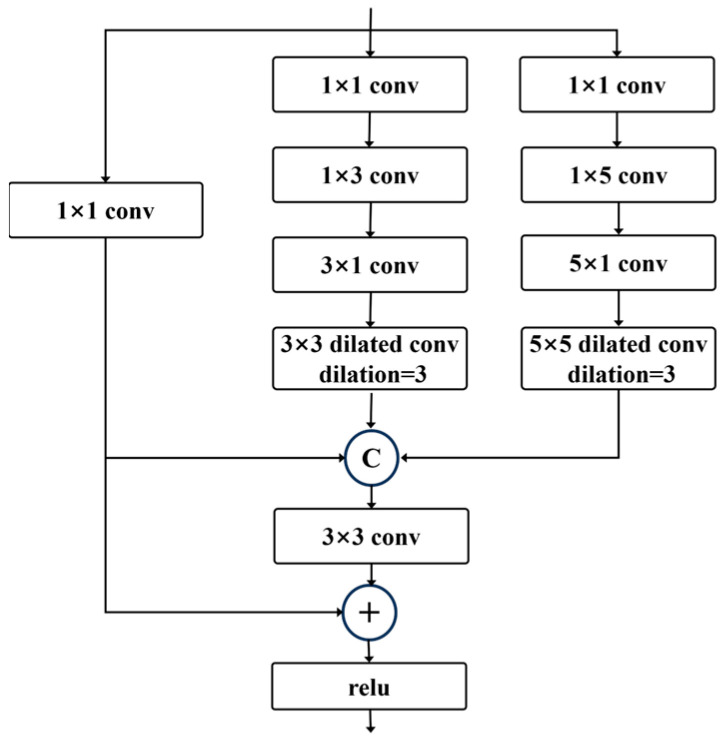
The structures of MSRF.

To better capture complex lesions with varied scales, we introduce the MSRF module for improved spatial representation. The MSRF employs a three-branch multi-scale convolutional architecture, with the computation path illustrated in Figure 3.

The base branch applies a 1 × 1 convolution directly to the input features to provide baseline information. The medium receptive field branch first applies a 1 × 1 convolution to the input features, followed by a combination of 1 × 3 and 3 × 1 convolutions, and then a 3 × 3 dilated convolution (dilation = 3) for feature extraction. The large receptive field branch first applies a 1 × 1 convolution to the input features, followed by a combination of 1 × 5 and 5 × 1 convolutions, and then a 5 × 5 dilated convolution (dilation = 3) to extract features at a larger scale. Finally, the features extracted from all branches are concatenated along the channel dimension and fused using a 3 × 3 convolution. Additionally, the input features are added to enhance the deep features.

### 3.4. Hierarchical Feature Transfer and Decoder

To fully utilize features from different levels, we propose an HFT mechanism, which enables effective transmission of deep semantic information to shallow layers. The first layer of the decoder directly receives $F_{4}^{SE}$ as input, ensuring that high-level semantic information is fully preserved during the decoding process. The intermediate layers of the decoder sequentially receive $F_{3}^{MSRF}$, $F_{2}^{MSRF}$, and $F_{1}^{MSRF}$ as inputs, with each feature passing through the MSRF module to enhance its multi-scale adaptability. The decoder inputs can be written as Equation (4):

(4) $$D_{k}=\left\{\begin{array}{ll} F_{4}^{SE}, & k=4,\\ Up_{k+1\to k}(D_{k+1})+F_{k}^{MSRF}, & k=3,2,1. \end{array}\right.$$

In the shallow layers of the decoder, we directly introduce the low-level features $T_{1}\in {\mathbb{R}}^{48\times 112\times 112}$. These features contain rich boundary and texture information, which helps to complement the fine structure of the lesion regions.

To further illustrate the complete data flow and the interaction among modules, the overall computation process of MSDTCN-Net is summarized in Algorithm 1.
**Algorithm 1** The PyTorch-Style Pseudo-Code of MSDTCN-Net**#Input:** X with shape [B, 3, 224, 224]**#Output:** Y with shape [B, n_classes, 224, 224]**#Operators:** CNX = ConvNeXt-Tiny encoder; RES = ResNet-34 encoderPE = positional embedding; DefT = Deformable Transformer encoderOut_k = Conv2d(256, Ck, kernel = 3, padding = 1) with (C0, C1, C2, C3, C4) = (48, 96, 192, 384, 768)Head = ConvTranspose2d (48 → 32, k = 4, s = 2, p = 1) → Conv2d(32 → 32, 3, p = 1) → Conv2d (32 → n_classes, 3, p = 1) ⊕ = element-wise addition; ‖ = channel concatenation1: C1..C4 = CNX(X)# (96, 56 × 56), (192, 28 × 28), (384, 14 × 14), (768, 7 × 7)2: R0..R4 = RES(X)# (64, 112 × 112), (64, 56 × 56), (128, 28 × 28), (256, 14 × 14), (512, 7 × 7)3: A0..A4 = Align1×1(R0)..Align1×1(R4)4: P0..P4 = PE(A0)..PE(A4); M0..M4 = zeros_bool_masks()5: mem, shapes, offsets = DefT ([A0..A4], [M0..M4], [P0..P4])6: O0..O4 = Split(mem)# each Oj: (256, Hj × Wj)7: T0..T4 = Out_0..Out_4(O0..O4)# (48, 112 × 112), (96, 56 × 56), (192, 28 × 28), (384, 14 × 14), (768, 7 × 7)8: F4_cat = Cat(C4‖T4); F4_se = SE(F4_cat); F4 = Conv1×1(F4_se)# (768,7 × 7)9: F3_cat = Cat(C3‖T3); F3_se = SE(F3_cat); F3 = Conv1×1(F3_se); F3m = MSRF(F3)#(384, 14 × 14)10: F2_cat = Cat(C2‖T2); F2_se = SE(F2_cat); F2 = Conv1×1(F2_se); F2m = MSRF(F2)#(192, 28 × 28)11: F1_cat = Cat(C1‖T1); F1_se = SE(F1_cat); F1 = Conv1×1(F1_se); F1m = MSRF(F1)#(96, 56 × 56)12: F0m = MSRF(T0)# (48, 112 × 112)13: D4 = F4;  D3 = UP4(D4)⊕F3m;  D2 = UP3(D3)⊕F2m;  D1 = UP2(D2)⊕F1m;  D0 = UP1(D1)⊕F0m14: Y = Head(D0)15: return Y

### 3.5. Loss Function

We adopt a hybrid loss function combining Binary Cross-Entropy Loss and Dice Loss to improve lesion recognition and maintain classification stability. The binary cross-entropy loss $L_{BCE}$ is defined in Equation (5):

(5) $$L_{BCE}=-\frac{1}{N}\sum_{i=1}^{N}\left[y_{i}\log({\hat{y}}_{i})+(1-y_{i})\log(1-{\hat{y}}_{i})\right]$$

where *N* is the total number of pixels in the image, $y_{i}$ is the true class of pixel *I*, and ${\hat{y}}_{i}$ is the predicted probability of the lesion for pixel *i*. The dice loss $L_{Dice}$ is defined in Equation (6):

(6) $$L_{Dice}=1-\frac{2\sum_{i=1}^{N}y_{i}{\hat{y}}_{i}+\epsilon }{\sum_{i=1}^{N}y_{i}+\sum_{i=1}^{N}{\hat{y}}_{i}+\epsilon }$$

where $y_{i}$ and ${\hat{y}}_{i}$ represent the true label and the model’s predicted probability, respectively. *ϵ* is a small smoothing term. Combining the advantages of $L_{BCE}$ and $L_{Dice}$, we adopt a weighted loss function to ensure a balance between overall classification performance and lesion area recognition capability [28], as shown in Equation (7):

(7) $$L=0.6\cdot L_{BCE}+0.4\cdot L_{Dice}$$

## 4. Experiments

### 4.1. Datasets

To evaluate the effectiveness of our method, we conducted experiments on four publicly available datasets. These four datasets are widely recognized in the field of skin lesion analysis and are extensively used for benchmarking and model evaluation. The first three datasets are ISIC 2016, ISIC 2017, and ISIC 2018, which are part of the dataset provided by the International Skin Imaging Collaboration (ISIC). The fourth dataset is the PH2 dataset, which was collected from the Dermatology Department at Pedro Hispano Hospital in Matosinhos, Portugal. In our experiments, the datasets were divided as follows:

(1) ISIC 2016: This dataset contains 1279 RGB skin lesion images with corresponding annotations. In the experiment, we randomly split the dataset, selecting 788 images for the training set, 112 images for the validation set, and 379 images for the test set.
(2) ISIC 2017: This dataset consists of 2750 annotated RGB images. The dataset was divided into 2000 training images, 150 validation images, and 600 test images.
(3) ISIC 2018: This dataset includes 2594 fully annotated RGB images. In the experiment, we randomly split the dataset, selecting 1815 images for the training set, 259 images for the validation set, and 520 images for the test set.
(4) PH2: This dataset contains 200 finely annotated RGB dermoscopic images. In the experiment, we randomly split the dataset, selecting 140 images for the training set, 20 images for the validation set, and 40 images for the test set.

It should be noted that ISIC 2016, ISIC 2017, and ISIC 2018 are all derived from the ISIC archive and may contain overlapping or visually similar lesions. In this study, we follow the common practice in existing work and do not exclude any images as duplicates.

### 4.2. Implementation Details

Our experiments were conducted in a Python 3.8 and PyTorch 2.0.0 environment, and implemented on an NVIDIA GeForce RTX 4090 GPU with 24 GB of VRAM. The resolution of all training, validation, and test images was uniformly adjusted to 224 × 224 to ensure consistent input dimensions. We selected ConvNeXt-Tiny and ResNet-34 as pre-trained models and Adam algorithm [34] to improve the model’s convergence speed and optimization stability. In the optimizer configuration, the learning rate was set to 0.001 to ensure stable weight updates. The momentum parameters, betas = (0.5, 0.999), were used to control the first and second moment estimates. The value of 0.5 reduces the inertia of the first moment estimate, making the optimizer more sensitive to changes in the current gradient compared to the traditional value of 0.9. This helps enhance training stability, while 0.999 smooths the second moment estimate to suppress gradient fluctuations. The weight decay was set to 0 to avoid the influence of additional L2 regularization on the model’s training process. We set the batch size to 4 during training. Throughout the training process, we monitored the model’s performance by calculating evaluation metrics on the validation set. If the Dice score on the validation set improved, we updated and saved the new weight file to ensure that the best model was used for subsequent testing.

### 4.3. Evaluation Metrics

We employed five commonly used metrics in skin lesion segmentation to evaluate the effectiveness of our proposed method: Accuracy (ACC), Intersection over Union (IoU), Dice coefficient (Dice), Sensitivity (SE), and Specificity (SP), as shown in Equations (8)–(12):

(8) $$ACC=\frac{TP+TN}{TP+TN+FP+FN}$$

(9) $$IoU=\frac{TP}{TP+FP+FN}$$

(10) $$Dice=\frac{2TP}{2TP+FP+FN}$$

(11) $$SE=\frac{TP}{TP+FN}$$

(12) $$SP=\frac{TN}{TN+FP}$$

where TP and TN represent the correctly identified skin lesion regions and background regions, respectively, FP refers to the background regions incorrectly identified as skin lesions, and FN represents the skin lesion regions incorrectly identified as background.

### 4.4. Ablation Studies

To evaluate the impact of each component in our proposed network architecture on the skin disease segmentation task, we designed and conducted 8 ablation experiments. The experiments were conducted using the ISIC 2017 dataset, with the same computational environment to ensure fairness of the results. The summarized experimental results are presented in Table 1. First, we used CE (ConvNeXt Encoder) as the baseline, which adopts a U-shaped architecture similar to U-Net, with ConvNeXt as the encoder. Then, we gradually added DE (Dual Encoder), SE (Squeeze-and-Excitation), HFT (Hierarchical Feature Transfer), and MSRF (Multi-Scale Receptive Field) to the network to construct several different experimental setups.

Compared to the CE method, the DE method achieved improvements of 1.44% and 1.01% in IoU and Dice, respectively. Compared to the CE + MSRF + HFT method, the IoU and Dice of the DE + MSRF + HFT method are improved by 3.03% and 2.44%, respectively.

Compared to the DE method, the DE + SE method showed improvements of 0.62% and 0.68% in IoU and Dice, respectively. Compared to the DE + MSRF + HFT method, the DE + SE + MSRF + HFT method achieved improvements of 1.07% and 0.63% in IoU and Dice, respectively.

Compared to the DE method, the DE + HFT method showed improvements of 3.83% and 2.70% in IoU and Dice, respectively. Compared to the DE + SE method, the DE + SE + HFT method improved IoU and Dice by 3.85% and 2.52%, respectively.

Compared to the DE + HFT method, the DE + MSRF + HFT method showed improvements of 0.88% and 0.72% in IoU and Dice, respectively. Compared to the DE + SE + HFT method, the DE + SE + MSRF + HFT method improved IoU and Dice by 1.31% and 0.85%, respectively.

### 4.5. Results on the ISIC 2016 Dataset

On the ISIC 2016 skin lesion segmentation dataset, we compared our method with 10 state-of-the-art segmentation methods. Table 2 summarizes the experimental results of each model in terms of ACC, IoU, Dice, SE, and SP. Our model achieved the highest scores in the three core metrics: ACC, IoU, and Dice, reaching 97.27%, 89.23%, and 94.02%, respectively. Notably, ACC improved by 0.63% over the second-best DEU-Net, IoU improved by 1.70% over the second-best ULFAC-Net, and Dice improved by 1.09% over ULFAC-Net, demonstrating the superiority of our method in terms of overall segmentation accuracy and region matching.

**Figure 4:**
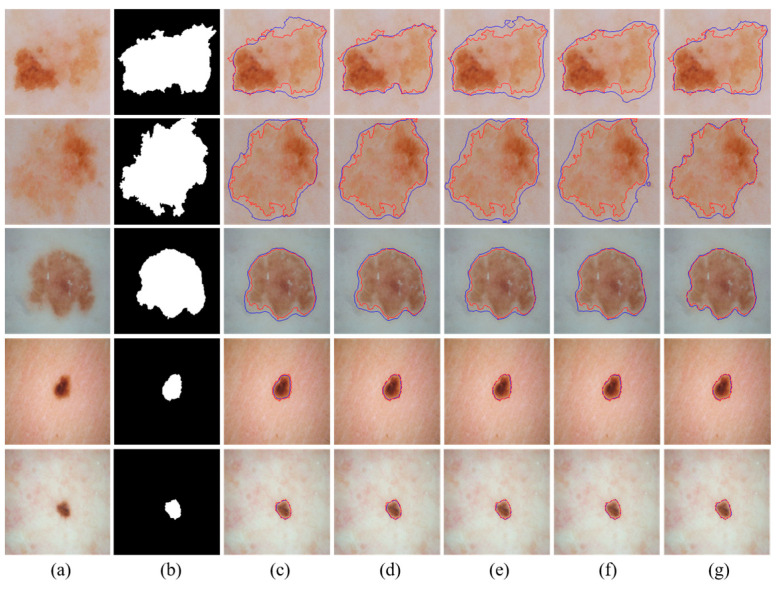
Visualization of segmentation results with different state-of-the-art methods on the ISIC 2016 dataset. (**a**) Input images. (**b**) Ground truth. (**c**) U-Net [6]. (**d**) FAT-Net [28]. (**e**) DCSAU-Net [23]. (**f**) TransAttUnet [26]. (**g**) Ours. The red contours represent the ground truth, while the blue contours indicate the segmentation results of the corresponding methods.

We visually compared the segmentation results of our proposed MSDTCN-Net with four other methods, as shown in Figure 4. The experimental results demonstrate that MSDTCN-Net outperforms the other methods in terms of boundary contour, segmentation accuracy, and region matching. When the lesion area has faint colors and low contrast, MSDTCN-Net still performs well in segmenting the lesion region.

**Figure 5:**
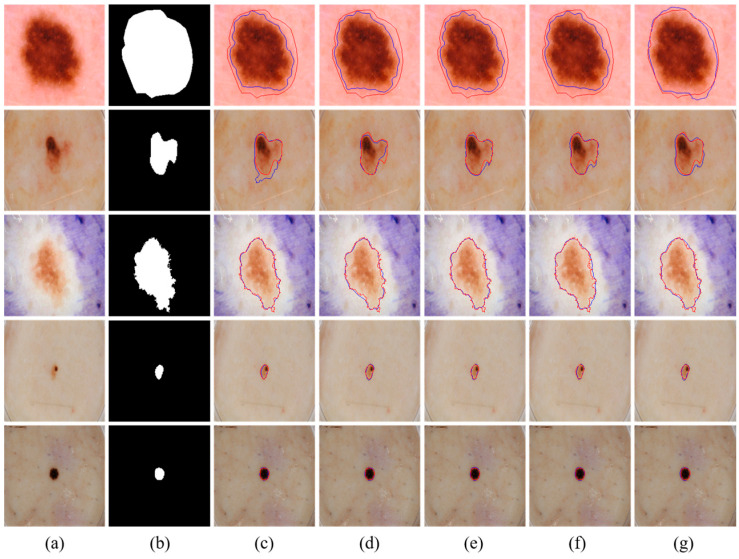
Visualization of segmentation results with different state-of-the-art methods on the ISIC 2018 dataset. (**a**) Input images. (**b**) Ground truth. (**c**) U-Net [6]. (**d**) FAT-Net [28]. (**e**) DCSAU-Net [23]. (**f**) TransAttUnet [26]. (**g**) Ours. The red contours represent the ground truth, while the blue contours indicate the segmentation results of the corresponding methods.

**Figure 6:**
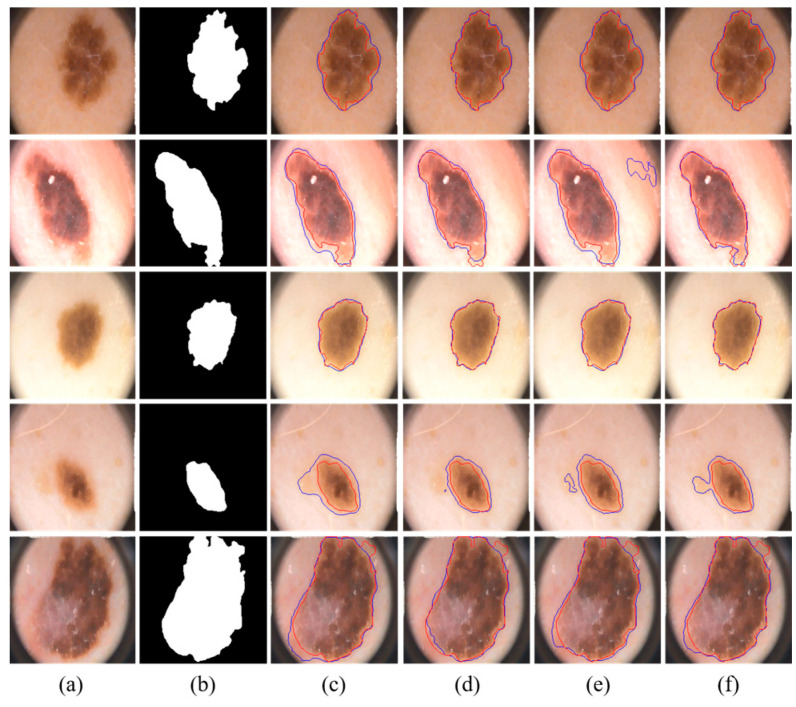
Visualization of segmentation results with different state-of-the-art methods on the PH2 dataset. (**a**) Input images. (**b**) Ground truth. (**c**) U-Net [6]. (**d**) DCSAU-Net [23]. (**e**) TransAttUnet [26]. (**f**) Ours. The red contours represent the ground truth, while the blue contours indicate the segmentation results of the corresponding methods.

It should be noted that the U-Net results reported in Table 2, Table 3 and Table 4 come from our retrained model. In contrast, the quantitative results of the other methods in these tables are directly taken from their original papers. In addition, all visualization results in Figure 4, Figure 5 and Figure 6 are generated from our retrained models.

### 4.6. Results on the ISIC 2018 Dataset

On the ISIC 2018 skin lesion segmentation dataset, we compared our method with 10 state-of-the-art segmentation methods. Table 3 summarizes the experimental results of each model on the ISIC 2018 skin lesion segmentation task. Our method achieved the highest scores on the two core metrics, IoU and Dice, with 85.01% and 90.92%, respectively. Specifically, IoU improved by 0.36% compared to the second-best model, ULFAC-Net, and Dice improved by 0.01%, indicating that our method is competitive in terms of overall segmentation accuracy and region matching.

We visually compared the segmentation results of our proposed MSDTCN-Net with four other methods, as shown in Figure 5. The experimental results show that MSDTCN-Net excels in both overall segmentation performance and local detail recovery. It maintains the consistency of lesion shapes and boundaries, effectively distinguishing details in complex and blurred edges.

### 4.7. Results on the PH2 Dataset

On the PH2 skin lesion segmentation dataset, we compared our method with 5 state-of-the-art segmentation methods. Table 4 summarizes the experimental results of each model on the PH2 skin lesion segmentation task. Our model achieved the highest scores in the three core metrics: ACC, IoU, and Dice, reaching 97.24%, 88.54%, and 93.74%, respectively. ACC improved by 0.2% over the second-best U-Net, IoU improved by 1.15% over the second-best SUTrans-NET, and Dice improved by 1.03% over CACDU-Net.

We visually compared the segmentation results of our proposed MSDTCN-Net with three other methods: U-Net, DCSAU-Net, and TransAttUnet, as shown in Figure 6. The experimental results show that MSDTCN-Net excels in delineating complex boundaries while effectively capturing small lesions with faint colors.

## 5. Discussion and Limitations

The encoder–decoder structure has been widely used in medical image segmentation tasks, achieving good performance on multiple datasets due to its hierarchical feature extraction and ability to progressively recover spatial information. However, existing methods still have limitations in capturing local details and modeling global context, which results in difficulties in achieving high-precision segmentation in complex lesion regions. To address these issues, we propose MSDTCN-Net, a dual-encoder architecture combining ConvNeXt and Deformable Transformer to capture local details and enhance global context modeling, improving lesion area perception. Our HFT mechanism optimizes feature flow during decoding, progressively fusing high-level semantics with low-level spatial details to improve complex boundary recovery. MSRF module, which combines multi-branch convolutions with dilated convolutions, improves the model’s adaptability to lesion areas of varying sizes. Ablation experiments on the ISIC 2017 dataset validated the contribution of each module. Experiments on the ISIC 2016, ISIC 2018, and PH2 datasets demonstrate strong generalization and robustness across various datasets and lesion types.

**Figure 7:**
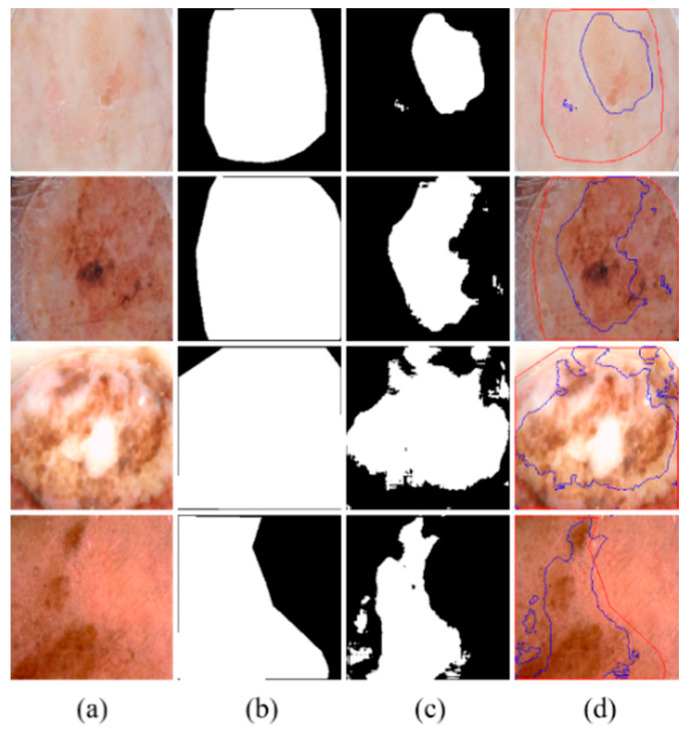
Visualization of challenging cases in skin lesion segmentation. (**a**) Input images. (**b**) Ground truth. (**c**) Predicted segmentation mask by the model. (**d**) Overlay of ground truth and prediction on the original image.

Although MSDTCN-Net has achieved significant performance improvements across multiple datasets, it still has certain limitations. When there is substantial color variation within the lesion region or when the contrast between the lesion and background is extremely low, the model may still struggle to accurately differentiate the lesion area, leading to boundary discrepancies (as shown in Figure 7). Despite the dual-encoder architecture effectively combining the advantages of CNN and Transformer, the model’s feature representation capability can still be limited in cases of low contrast or uneven texture, which may hinder accurate segmentation in such challenging scenarios.

Beyond these accuracy-side limitations under challenging imaging conditions, there is also a cost-side constraint: the dual-encoder design introduces computational overhead. Incorporating a Deformable Transformer increases parameters relative to single-encoder baselines, which raises resource consumption during training and inference. However, this trade-off yields accuracy benefits. In practice, efficiency can be improved via model compression and architectural simplification (e.g., reducing Transformer encoder depth), seeking a better balance between performance and cost.

From a clinical deployment standpoint, the modular design of MSDTCN-Net enables flexible adoption. Lightweight variants are suitable for hospital servers or edge devices for near–real-time screening, whereas the full model can support offline, high-precision analysis. Future work will prioritize improving computational efficiency and exploring adaptive inference schemes to balance segmentation accuracy, latency, and hardware constraints.

## 6. Conclusions

In this study, we propose MSDTCN-Net, a model based on the dual-encoder architecture combining ConvNeXt and Deformable Transformer, aimed at improving the accuracy of skin lesion segmentation, particularly in regions with complex boundaries and irregular lesion shapes. The proposed HFT mechanism passes multi-scale features layer by layer, ensuring effective interaction between high-level semantic information and low-level spatial details, thereby enhancing the decoder’s ability to recover lesion boundaries. The designed MSRF module combines multi-branch convolutions and dilated convolutions, boosting the model’s adaptability to lesions of varying scales and ensuring stable segmentation performance even in cases of complex morphology or fuzzy boundaries. To validate the effectiveness of MSDTCN-Net, we conducted extensive experiments on four publicly available skin lesion datasets: ISIC 2016, ISIC 2017, ISIC 2018, and PH2. The experimental results demonstrate that our method exhibits competitive segmentation performance across key metrics such as IoU, Dice, and ACC, surpassing existing state-of-the-art segmentation methods on multiple datasets. Additionally, intuitive visual analysis further confirms MSDTCN-Net’s capability in segmenting complex boundary regions.

## Figures and Tables

**Table 1:** Statistical comparison of the ablation study on different components in MSDTCN-Net.

Method	ACC (%)	IoU (%)	Dice (%)	SE (%)	SP (%)	Params (M)	Infer-T (ms)
CE	93.25	74.14	83.50	80.63	97.45	55.95	25.91
DE	93.80	75.58	84.51	84.74	96.86	91.49	52.00
DE + SE	94.05	76.20	85.19	87.00	95.38	91.78	51.71
DE + HFT	94.72	79.41	87.21	84.88	97.22	91.88	52.11
CE + MSRF + HFT	93.97	77.26	85.49	81.90	97.86	71.45	28.49
DE + MSRF + HFT	94.96	80.29	87.93	86.01	96.99	107.56	55.16
DE + SE + HFT	94.94	80.05	87.71	86.30	96.73	92.27	52.30
DE + SE + MSRF + HFT (Ours)	95.41	81.36	88.56	86.19	97.51	107.95	56.37

**Table 2:** Comparison of performance across state-of-the-art methods on the ISIC 2016 dataset.

Method	Year	ACC (%)	IoU (%)	Dice (%)	SE (%)	SP (%)
U-Net [6]	2015	95.25	84.69	91.12	89.93	**97.86**
FAT-Net [28]	2022	96.04	85.30	91.59	92.59	96.02
Pact-Net [30]	2023	95.57	86.95	92.56	**94.98**	95.75
DEU-Net [29]	2023	96.64	87.48	92.90	92.81	97.40
ULFAC-Net [35]	2023	96.35	87.53	92.93	93.69	96.56
GFANet [36]	2023	96.04	85.92	91.78	92.95	97.25
DCSAU-Net [23]	2023	95.86	85.85	91.68	91.92	97.04
TransAttUnet [26]	2024	96.30	85.98	91.79	92.15	97.12
SUTrans-NET [27]	2024	95.90	85.48	91.43	93.66	96.19
SLP-Net [37]	2025	95.70	84.43	91.12	91.45	96.94
Ours	-	**97.27**	**89.23**	**94.02**	94.00	97.41

**Table 3:** Comparison of performance across state-of-the-art methods on the ISIC 2018 dataset.

Method	Year	ACC (%)	IoU (%)	Dice (%)	SE (%)	SP (%)
U-Net [6]	2015	95.42	80.68	87.93	86.54	**98.22**
FAT-Net [28]	2022	95.78	82.02	89.03	91.00	96.99
ULFAC-Net [35]	2023	96.52	84.65	90.91	91.96	97.56
Pact-Net [30]	2023	**96.91**	84.32	90.75	91.87	97.27
DEU-Net [29]	2023	96.57	84.44	90.81	**92.40**	97.51
GFANet [36]	2023	96.29	83.66	90.13	90.75	97.79
DCSAU-Net [23]	2023	96.02	82.41	89.05	89.02	97.68
TransAttUnet [26]	2024	95.92	82.29	88.98	88.82	97.78
SUTrans-NET [27]	2024	93.33	81.63	88.58	91.93	94.02
DSU-Net [38]	2025	94.31	83.43	90.04	92.22	96.14
Ours	-	96.52	**85.01**	**90.92**	90.21	97.66

**Table 4:** Comparison of performance across state-of-the-art methods on the PH2 dataset.

Method	Year	ACC (%)	IoU (%)	Dice (%)	SE (%)	SP (%)
U-Net [6]	2015	97.04	86.73	92.58	98.68	96.37
CACDU-Net [39]	2023	95.04	86.52	92.71	97.06	94.09
DCSAU-Net [23]	2023	96.17	85.99	91.23	96.40	**96.87**
TransAttUnet [26]	2024	97.02	86.96	92.70	**98.90**	96.17
SUTrans-NET [27]	2024	94.59	87.39	92.56	96.71	91.77
Ours	-	**97.24**	**88.54**	**93.74**	97.77	96.36

## Data Availability

The data presented in this study are openly available in ISIC at https://challenge.isic-archive.com/data/ (accessed on 17 February 2025). The data presented in this study are openly available in PH² database at https://www.fc.up.pt/addi/ph2%20database.html (accessed on 19 February 2025).
